# What, where, and how: Regulation of translation and the translational landscape in plants

**DOI:** 10.1093/plcell/koad197

**Published:** 2023-07-12

**Authors:** Hsin-Yen Larry Wu, Joey Jen, Polly Yingshan Hsu

**Affiliations:** Department of Biochemistry and Molecular Biology, Michigan State University, East Lansing, MI 48824, USA; Department of Biochemistry and Molecular Biology, Michigan State University, East Lansing, MI 48824, USA; Department of Biochemistry and Molecular Biology, Michigan State University, East Lansing, MI 48824, USA

## Abstract

Translation is a crucial step in gene expression and plays a vital role in regulating various aspects of plant development and environmental responses. It is a dynamic and complex program that involves interactions between mRNAs, transfer RNAs, and the ribosome machinery through both cis- and trans-regulation while integrating internal and external signals. Translational control can act in a global (transcriptome-wide) or mRNA-specific manner. Recent advances in genome-wide techniques, particularly ribosome profiling and proteomics, have led to numerous exciting discoveries in both global and mRNA-specific translation. In this review, we aim to provide a “primer” that introduces readers to this fascinating yet complex cellular process and provide a big picture of how essential components connect within the network. We begin with an overview of mRNA translation, followed by a discussion of the experimental approaches and recent findings in the field, focusing on unannotated translation events and translational control through cis-regulatory elements on mRNAs and trans-acting factors, as well as signaling networks through 3 conserved translational regulators TOR, SnRK1, and GCN2. Finally, we briefly touch on the spatial regulation of mRNAs in translational control. Here, we focus on cytosolic mRNAs; translation in organelles and viruses is not covered in this review.

## Teaser

Overview of translational regulation and new translation events in plants.

## Introduction

### Basics of mRNA translation

Eukaryotic gene expression begins with the transcription of genes in the nucleus. The resulting transcripts are conceptually classified into protein-coding RNAs (or mRNAs) and noncoding RNAs, although translation from some noncoding RNAs has been reported (see section on ‘Unannotated translation events’). Following transcription, the primary transcripts or pre-mRNAs undergo processing, including the addition of a 5′ cap and a 3′ poly-A tail and splicing. Once the mature mRNAs are transported out of the nucleus, they are generally ready for translation. However, mRNAs may be degraded or stored in some circumstances, preventing them from being translated.

The major players in translation include mRNAs, ribosomes, transfer RNAs (tRNAs), and translation factors involved in different phases of translation. Below we briefly describe the main steps of translation and core translation factors. For detailed composition of the translation machinery in plants, including ribosomal proteins and translation factors, we refer readers to this comprehensive review (Browning and Bailey-Serres 2015).

**Figure 1.**
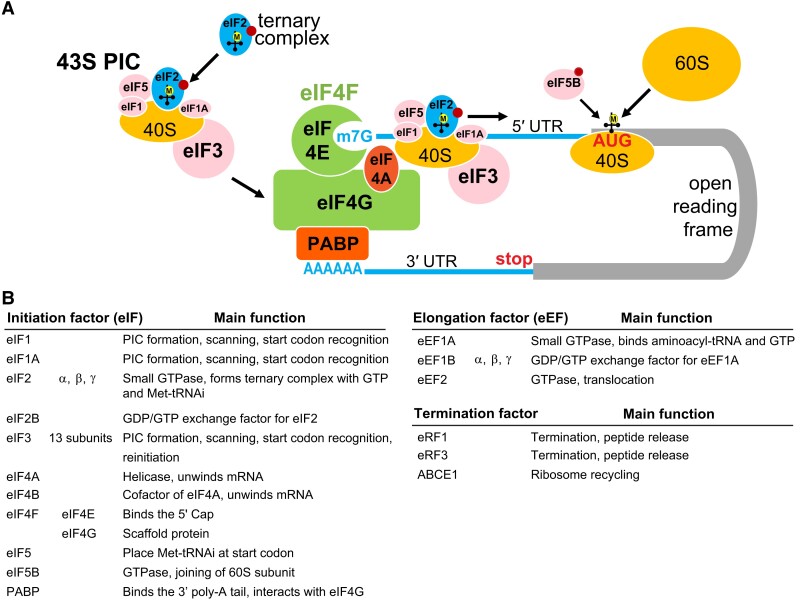
Overview of eukaryotic translation initiation complexes and functions of core translation factors. **A)** Eukaryotic mRNAs typically have a 5′ m7G cap and a 3′ poly-A tail. Translation initiation is controlled by 2 large protein complexes, including the eIF4F complex and the 43S PIC. eIF4F consists of eIF4E (cap binding) and eIF4G (a scaffold protein). The 43S PIC consists of the 40S ribosomal subunit, the ternary complex (eIF2, GTP, Met-tRNA_i_), eIF1, eIF1A, eIF3, and eIF5. The eIF4F complex binds to the cap and recruits the 43S PIC, which then scans the 5′ UTR until a start codon is identified. Subsequently, most initiation factors dissociate, and the 60S ribosomal subunit joins. GTP is shown in dark red circles. The figure was adapted from (Castellano and Merchante 2021; Passmore and Coller 2022). **B)** Summary of core translation factors involved in canonical initiation, elongation, and termination in Arabidopsis. The information was simplified and adapted from Browning and Bailey-Serres (2015) and Raabe et al. (2019).

Eukaryotic mRNAs typically have a 5′ cap (7-methylguanosine) and a 3′ poly-A tail (Fig. 1A: an overview of translation initiation; Fig. 1B: a summary of core translation factors). The cap serves as an anchor for translational initiation. Cap-dependent translation initiation is governed by 2 large protein complexes: the eIF4F complex (consisting of eukaryotic initiation factor 4E (eIF4E) and eIF4G) and the 43S pre-initiation complex (PIC) (consisting of the 40S ribosomal small subunit, ternary complex: eIF2/GTP/Met-tRNA_i_, eIF1, eIF1A, eIF3, and eIF5). First, the 5′ cap is recognized by eIF4E, which forms a complex with eIF4G, a large scaffold protein that interacts with the RNA helicase eIF4A. The 43S PIC is then recruited to the cap through the interaction between multimeric eIF3 and eIF4G. eIF4G also interacts with poly-A binding protein (PABP), which binds to the poly-A tail at the 3′ end of the mRNA. Thus, the 5′ and 3′ ends of the mRNAs are connected, forming a circular structure. The circularization of mRNAs enhances their efficient translation, facilitates ribosome recycling, and increases mRNA stability by protecting mRNAs from degradation via exonucleases (reviewed in Browning and Bailey-Serres 2015; Passmore and Coller 2022).

Translation can be divided into 3 phases: initiation, elongation, and termination. Translation initiation is considered the key point of regulation and is the most well studied of these 3 phases. Plants, like other eukaryotes, use the scanning model for translation initiation (Fig. 1A). After being recruited to the cap, the 43S PIC scans along the 5′ leader sequence (i.e. the region upstream of the main ORF, also commonly called 5′ UTR, although extensive translation may occur in this region; see section on ‘Unannotated translation events’) until an optimal start codon is identified. Then, the 60S ribosomal large subunit is recruited, with the Met-tRNA_i_ positioned at the peptidyl site (P-site) of the ribosome, and the codon on the mRNA and anti-codon on the tRNA base pair. New charged tRNAs corresponding to the next codons then join at the aminoacyl site (A-site). The amino acids at the P-site and A-site form a peptide bond. Subsequently, the amino acid (or the growing peptide) at the P-site is transferred to the amino acid at the A-site. Next, the ribosome translocates by shifting 3 nucleotides (3 nts; 1 codon). As a result, the uncharged tRNA is now located at the exit site, and the tRNA carrying the peptide is at the P-site, which makes the A-site available for the incoming charged tRNAs. This cycle repeats until a stop codon is encountered at the A-site, where release factors, rather than a charged tRNA, join the A-site and trigger translation termination. Subsequently, the ribosome subunits dissociate from the mRNA and are recycled for additional rounds of translation (reviewed in Schuller and Green 2018).

### Unique features of plant mRNA translation

Although the machinery for plant mRNA translation is largely similar to that of other eukaryotes, several unique characteristics have evolved in plants. In addition to the canonical eIF4F complex, consisting of eIF4E and eIF4G, plants possess eIFiso4F complexes consisting of eIFiso4E and eIFiso4G (encoded by *eIFiso4G1* and *eIFiso4G2* in Arabidopsis [*Arabidopsis thaliana*]). eIFiso4G is deeply conserved throughout plant lineages, whereas eIFiso4E is present only in flowering plants. The eIFiso4F complex is smaller compared with eIF4F complex (∼80 kD vs ∼180 kD) but more abundant (Patrick and Browning 2012). eIF4G and eIFiso4G have different mRNA sequence preferences and interact differently with various PABP isoforms (Gallie and Browning 2001; Gallie 2018); thus, these complexes may favor specific sets of mRNAs and control distinct physiological responses. For example, eIFiso4G has been shown to prefer mRNAs with unstructured sequences, which are characteristics of mRNAs that are preferentially translated under hypoxia (Gallie and Browning 2001; Branco-Price et al. 2005). More recently, eIFiso4G has been reported to control specific mRNAs involved in chloroplast functions, hypoxia, and abscisic acid (ABA) signaling (Bi et al. 2019; Cho et al. 2019; Lellis et al. 2019).

Plant ribosomal proteins have also expanded due to multiple rounds of genome duplication. In Arabidopsis, there are 80 ribosomal protein (RP) families, and each RP is encoded by 2 to 6 genes (Barakat et al. 2001; Browning and Bailey-Serres 2015; Scarpin et al. 2023). The large number of potential combinations of these RPs in ribosomes is proposed to provide heterogeneity to ribosome properties and enable specific mRNA translation in response to various internal and external cues (Browning and Bailey-Serres 2015).

### Genome-wide techniques to study translation

As translation involves mRNAs, which can be amplified and sequenced, much of our knowledge of translational regulation has expanded thanks to the development of next-generation sequencing. Below we discuss 3 common genome-wide techniques for studying translation. For other techniques, we refer readers to a comprehensive review (Mazzoni-Putman and Stepanova 2018).

**Figure 2.**
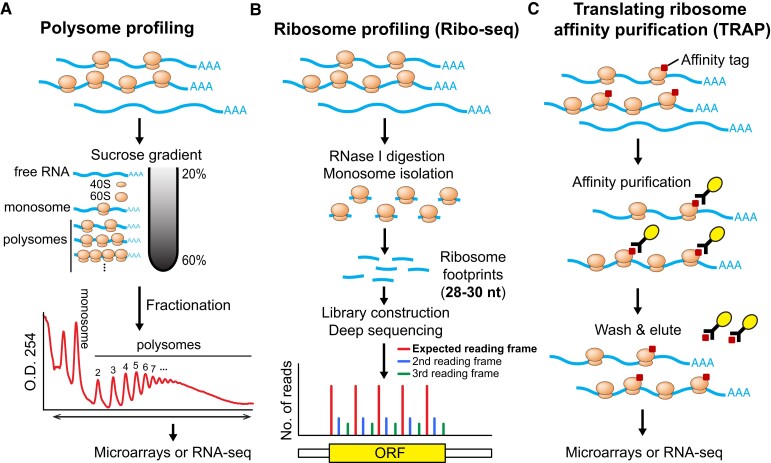
Common genomic techniques for studying translation. **A)** Polysome profiling uses a sucrose gradient to separate mRNAs associated with different numbers of ribosomes. The resulting mRNA populations corresponding to various translational activities can be fractionated and subjected to microarray or RNA-seq for identifying and quantifying the mRNAs. **B)** Ribo-seq uses ribonuclease digestion to obtain ribosome footprints. Sequencing of ribosome footprints reveals the precise position and quantity of ribosomes on mRNAs globally. High-quality datasets display a strong 3-nt periodicity that corresponds to ribosomes translocating by 3 nt per codon within coding regions. **C)** TRAP uses antibodies to isolate a subpopulation of ribosomes by expressing an epitope-tagged ribosomal large subunit protein driven by either a constitutive or a tissue-specific promoter. The mRNAs associated with the purified ribosomes can be subjected to microarray or RNA-seq for identifying and quantifying the mRNAs.

The first genomic technique used to study translation is polysome profiling (Fig. 2A), which involves separating mRNAs bound by various numbers of ribosomes through a sucrose gradient by ultracentrifugation. The distribution of specific mRNAs in different fractions can be quantified using reverse-transcription PCR, microarrays, or RNA-sequencing (RNA-seq) to estimate their relative translational activity. Although this technique is powerful and has allowed countless discoveries (e.g. Coate et al. 2014; Missra et al. 2015; Bai et al. 2017a; Zhang et al. 2017), polysome profiling cannot reveal the exact ribosome occupancy on mRNAs, and the resolution of polysome separation typically decreases as the number of bound ribosomes increases (Fig. 2A).

The second technique, termed ribosome profiling (a.k.a. Ribo-seq; Fig. 2B), solves the limitation of polysome profiling. Ribo-seq involves ribonuclease digestion of ribosome-bound mRNAs and sequencing of the resulting ribosome-protected mRNA fragments (i.e. ribosome footprints), typically 28 to 30 nts in length (Ingolia et al. 2009; Brar and Weissman 2015). The sequencing of these ribosome footprints reveals the precise quantity and position of ribosome occupancy on mRNAs throughout the transcriptome. Translation efficiency can be inferred by normalizing the Ribo-seq to RNA-seq read density. Moreover, in high-quality Ribo-seq data, ribosome footprints display strong 3-nt periodicity, consistent with translating ribosomes moving every 3 nts along coding regions. This periodic property can be used to identify actively translated regions (Ingolia et al. 2009). The first ribosome profiling studies in Arabidopsis were reported a decade ago; one was applied to study translational control of light responses (Liu et al. 2013), and a variation of the technique was applied to study chloroplast translation (Zoschke et al. 2013). Optimizing the resolution of Ribo-seq led to the discovery of previously unannotated translated ORFs, which dramatically expanded the landscape of the Arabidopsis translatome and improved genome annotation (Hsu et al. 2016). Ribo-seq has been used to study translational responses to many environmental stimuli, such as hypoxia, hormonal responses, pathogen responses, and phosphate deficiency in Arabidopsis (Juntawong et al. 2014; Merchante et al. 2015; Bazin et al. 2017; Xu et al. 2017a) and to characterize the translatomes in crops such as maize (*Zea mays*), soybean (*Glycine max*), tomato (*Solanum lycopersicum*), rice (*Oryza sativa*), sorghum (*Sorghum bicolor*), and wheat (*Triticum aestivum*) (Lei et al. 2015; Chotewutmontri and Barkan 2016; Shamimuzzaman and Vodkin 2018; Wu et al. 2019; Li and Liu 2020; Zhu et al. 2021; Sotta et al. 2022; Guo et al. 2023b). However, the short length of ribosome footprints limits their mapping capability. Therefore, it is challenging to implement Ribo-seq for polyploid genomes where short reads are less likely to be uniquely mapped (Jiang et al. 2022). Nevertheless, Ribo-seq has been recently carried out in hexaploid bread wheat (Guo et al. 2023b).

The third technique, translating ribosome affinity purification (TRAP; Fig. 2C), involves the expression of an epitope-tagged ribosomal protein (usually a large ribosomal subunit protein to ensure the capture of fully assembled ribosomes), followed by affinity purification of the tagged ribosomes and associated mRNAs using antibodies. The mRNAs associated with translating ribosomes can then be identified and quantified by microarrays or RNA-seq (Inada et al. 2002; Mustroph et al. 2009; Juntawong et al. 2014). Using a constitutive promoter to express the tagged ribosomal protein, TRAP-seq has been applied to study translational responses to different treatments in several species (Juntawong et al. 2014; Bazin et al. 2017; Meteignier et al. 2017; Reynoso et al. 2019; Bonnot and Nagel 2021). When combined with tissue-specific promoters, TRAP enables the study of tissue-specific translatomes (Mustroph et al. 2009; Traubenik et al. 2020; Kajala et al. 2021; Reynoso et al. 2022). It should be noted that TRAP requires generating stable transgenic plants, and the accumulation of tagged ribosomal proteins may alter ribosome properties and physiology (Zhao et al. 2019a). Although TRAP-seq can reveal mRNAs engaged in translation within specific tissue types, it is difficult to estimate translation efficiency (average ribosome number per mRNA) because it is technically challenging to quantify mRNA levels from the exact same tissue used to produce the TRAP-seq data. Although, to our knowledge, there are no reports in plants yet, it is possible to combine tissue-specific TRAP with ribosome footprinting (Gonzalez et al. 2014; Chen and Dickman 2017; Doroudgar et al. 2019) to quantify ribosome density and study actively translated ORFs on tissue-specific mRNAs.

## Unannotated translation events

The above sequencing techniques, especially Ribo-seq, have transformed our view of translational landscapes. In addition to refining previously annotated ORF models, Ribo-seq has led to 3 major discoveries in the translatomes: (1) the translation of small ORFs (sORFs) in RNAs presumed to be noncoding, (2) the widespread presence of upstream ORFs (uORFs) on protein-coding mRNAs, and (3) the usage of alternative or non-AUG start sites. Unlike canonical protein-coding genes, the act of translating these new ORFs, specifically sORFs and uORFs, may serve a regulatory function, even if the resulting protein products do not play a direct role in regulation (see below).

### Newly discovered translated sORFs among presumed noncoding RNAs

To minimize false discoveries, computational genome annotations typically assume that coding sequences are longer than 100 codons. This assumption means that transcripts encoding small proteins can be overlooked and annotated as noncoding RNAs (Basrai et al. 1997). Based on the 3-nt periodicity corresponding to ribosomes translocating 3 nt per codon, dozens or hundreds of translated sORFs within annotated noncoding RNAs or previously unannotated transcripts have been identified in Arabidopsis, tomato, maize, and wheat (Hsu et al. 2016; Wu et al. 2019; Kurihara et al. 2020; Liang et al. 2021; Palos et al. 2022; Guo et al. 2023b). Similarly, an alternative approach based on ribosome dissociation from mRNAs upon translation termination identified over 200 translated sORFs in Arabidopsis (Bazin et al. 2017). The translation efficiency of sORF mRNAs in tomato is indistinguishable from that of canonical protein-coding genes, suggesting that most of these sORF transcripts are likely to be true protein-coding RNAs (Wu et al. 2019). Epitope-tagging followed by western blots and mass spectrometry–based proteomic analysis confirmed that at least some of these sORFs produce stable proteins in planta (Hsu et al. 2016; Bazin et al. 2017; Wu et al. 2019).

Although most of these sORF mRNAs were likely mis-annotated as noncoding RNAs, surprisingly, some well-characterized noncoding RNAs also contain translated sORFs. The best documented examples are the primary transcripts of trans-acting small interfering RNAs (tasiRNAs). The biogenesis of tasiRNAs involves microRNA (miRNA) cleavage of primary *TAS* transcripts, which are then converted to double-stranded RNAs (dsRNAs) and processed into 21- or 22-nt tasiRNAs. Arabidopsis has 4 *TAS* gene families (*TAS1* to *TAS4*). The primary *TAS3* transcript contains a 50-amino acid (aa) sORF upstream of the first miRNA390 binding site; strong 3-nt periodicity of Ribo-seq reads was unambiguously detected in this sORF, supporting that it is actively translated (Hsu et al. 2016; Hsu and Benfey 2018). The ribosome occupancy on the *TAS3* sORF is also supported by a genome-wide study of 5′ truncated mRNA ends (Hou et al. 2016). By contrast, the primary *TAS2* transcript has a 57-aa sORF that contains a miRNA173 binding site. This sORF was shown to be translated in vitro, and introducing a premature stop codon to this sORF reduces tasiRNA levels. These results suggest that the translation and position of the sORF relative to the miRNA binding site are critical for tasiRNA biogenesis (Yoshikawa et al. 2016). In addition, primary *TAS1A/B/C*, *TAS2*, and *TAS3* transcripts are associated with membrane-bound polysomes, suggesting that they are translated by endoplasmic reticulum (ER)-localized ribosomes (Li et al. 2016). A recent study further demonstrated that the dsRNA-binding protein SUPPRESSOR OF GENE SILENCING 3 (SGS3) regulates ribosome stalling at the sORFs upstream of the miRNA binding sites on *TAS3* and *TAS1C* by forming a complex with ARGONAUTE (AGO) and the relevant miRNA (Iwakawa et al. 2021).

Another surprising example of noncoding RNA translation was found in the primary transcripts of miRNAs. The first study reported that *pri-miR165a* encodes an 18-aa sORF in Arabidopsis and *pri-miR171b* encodes a 9-aa sORF in *Medicago truncatula* (Lauressergues et al. 2015). Application of synthetic peptides corresponding to these sORFs led to an increase in *pri-miRNA* levels (Lauressergues et al. 2015; Sharma et al. 2020). However, the translation levels of these sORFs appear to be low or condition dependent, as no significant ribosome footprints in these sORF regions have been detected in available Ribo-seq datasets (Hsu and Benfey 2018).

### Prevalent uORFs on mRNAs

**Figure 3.**
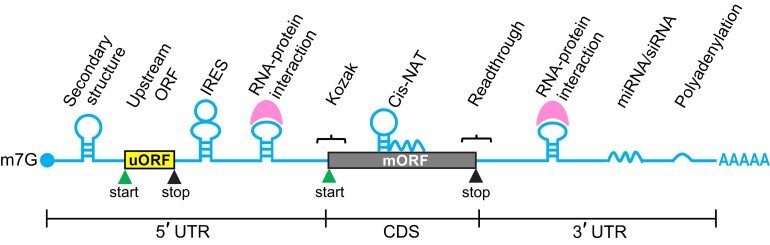
Common cis-regulatory elements or mechanisms contributing to translational control in mRNAs. Cis-regulatory elements or associated mechanisms commonly present in the 5′ UTR, the mORF, or 3′ UTR are presented. The figure was adapted from Liu and Qian (2014).

Conventionally, it was assumed that 1 mRNA produces 1 protein from the longest ORF. However, when the 43S PIC scans along the 5′ UTR searching for the optimal start site (usually AUG), it may encounter 1 or multiple smaller ORFs upstream of the annotated ORFs (thus the name upstream ORFs). As a result, the ribosome may translate the uORF before reaching the longest/annotated ORF (main ORF; mORF) (Fig. 3). Ribo-seq analysis has revealed numerous translated uORFs in plants, for example, 2,093 in Arabidopsis, 1,329 in tomato, 2,558 in maize, and 1,254 in wheat (Lei et al. 2015; Wu et al. 2019; Wu and Hsu 2022; Guo et al. 2023b). The identification of these uORFs suggests that the translation of their downstream mORFs is likely repressed by the uORFs (see section ‘Translational regulation through mRNA features – uORFs’ for details).

### Alternative or non-AUG translation start sites

Like uORFs, the 43S PIC may consider codons different from the annotated start site to initiate translation, which can lead to the production of proteins different from the annotated ORFs through the use of an alternative AUG or a near-cognate start site that differs from AUG by 1 nucleotide. Recently, a specialized Ribo-seq termed translation initiation site sequencing (TI-seq) has been applied in plants to study translation initiation and amino-terminal (N-terminal) proteomics (Willems et al. 2017; Li and Liu 2020). TI-seq uses the translation inhibitor lactimidomycin to capture initiating ribosomes, in contrast to regular Ribo-seq that uses cycloheximide, which captures both initiating and elongating ribosomes. Note that even snap freezing in liquid nitrogen during sample harvest can capture many elongating ribosomes (Douka et al. 2022). Thus, a technical challenge with TI-seq is to ensure efficient runoff of elongating ribosomes to avoid false positives. Therefore, stringent criteria and careful follow-up validation are necessary (Gao et al. 2015; Li and Liu 2020). Studies in Arabidopsis cultured cells and tomato leaves have revealed that alternative or near-cognate translation start sites are prevalent. The most common near-cognate codon is CUG, whereas AAG and AGG are the least used (Willems et al. 2017; Li and Liu 2020). Many alternative translation start sites identified in plants to date are in frame with the corresponding annotated ORF, resulting in changes in the protein N-terminal sequences (Willems et al. 2017; Wu et al. 2019; Li and Liu 2020). Follow-up experiments have demonstrated that several alternative or non-AUG translation start sites lead to different protein subcellular localizations (Li and Liu 2020).

## Translational regulation through mRNA features

Translational control can be classified conceptually into cis- and trans-regulation. An mRNA molecule may contain specific cis-regulatory elements that contribute to translational control. Many cis-regulatory elements have been found within the 5′ UTR, 3′ UTR, or surrounding the start or stop codons of mRNAs (Fig. 3). Some of these cis-regulatory elements are known to be bound or regulated by specific RNA-binding proteins (i.e. trans-acting factors). In this section, we will discuss gene-specific cis-regulatory elements involved in translational control and their corresponding RNA-binding proteins, if known. In the next section ‘Translational regulation through modulation of translation factors and master translational regulators’, we will focus on translation factors, mechanisms, or signaling that can affect global translation.

### Kozak

During its search for the start codon, the 43S PIC may encounter multiple AUG and near-cognate codons present in the 5′ UTR. How does the ribosome decide where to start? An optimal start codon typically possesses a consensus sequence flanking the AUG known as the Kozak consensus sequence (Kozak 1981). In plants, as well as in other eukaryotes, the Kozak consensus sequence is 5′-A/GNNAUGG- 3′, meaning that the upstream third nt (−3) of the AUG is enriched for A or G, and the downstream fourth nt (+4) is enriched for G (Lütcke et al. 1987; Nakagawa et al. 2008). Analysis of actively translated ORFs from Ribo-seq data in Arabidopsis, tomato, maize, and sorghum has confirmed the conserved sequence bias toward −3/+4 positions (Liu et al. 2013; Lei et al. 2015; Wu et al. 2019; Sotta et al. 2022). Thus, mRNA sequences containing an optimal Kozak consensus sequence are expected to have better translation efficiency.

### uORF

#### uORF properties

**Figure 4.**
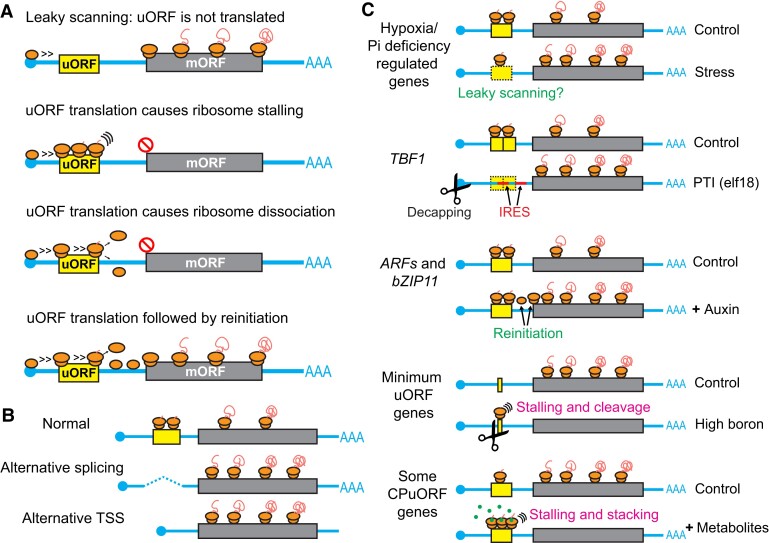
Overview of uORF regulation and expected mORF translation levels. **A)** Effects of uORFs on mORF translation may include leaky scanning, ribosome stalling, ribosome dissociation, and re-initiation. **B)** Mechanisms that regulate the presence of uORFs in mRNA sequences. uORFs may be omitted through alternative splicing or using an alternative transcription start site of mRNAs. uORFs are shown in yellow boxes; mORFs are shown in grey boxes. **C)** Examples of uORF regulation under different environmental conditions and expected mORF translation levels. mRNA degradation is shown by scissors. Metabolites are shown by green dots. PTI: pattern-triggered immunity.

As 30% to 70% of plant genes contain potential AUG-initiated uORFs (Zhang et al. 2021), uORFs represent a common mechanism for translational control. If a uORF is translated, it may lead to ribosome stalling or dissociation before the ribosome reaches the mORF, resulting in repression of mORF translation (Fig. 4A). Moreover, when a uORF is translated, the transcript will appear to have a long 3′ UTR, which is expected to activate nonsense-mediated decay (NMD), a conserved mechanism to degrade aberrant mRNAs with a premature termination codon (Kurosaki et al. 2019). Although evidence in animals supports the idea that uORF translation triggers NMD (Hurt et al. 2013; Chew et al. 2016; Johnstone et al. 2016; Baird et al. 2018), whether uORFs trigger NMD in plants is still under debate, particularly with new evidence in Arabidopsis challenging this idea (Wu and Hsu 2022).

The mORF downstream of a uORF may still get translated via leaky scanning or re-initiation (Fig. 4A). Leaky scanning occurs when the 43S PIC passes a uORF without translating it, and, thus, not all predicted uORFs are translated. In the case of re-initiation, after the uORF is translated, the ribosomal small subunit remains attached to the mRNA and continues scanning the 5′ UTR, ultimately translating the mORF. Factors that determine whether a uORF is translated or the efficiency of re-initiation remain areas of great interest.

uORFs are enriched in the transcripts of genes encoding transcription factors and protein kinases (Kim et al. 2007; Wu and Hsu 2022) and are present in the transcripts of numerous metabolic genes (reviewed in Van Der Horst et al. 2020). uORFs are also associated with allelic variation and agricultural traits (Gage et al. 2022; Guo et al. 2022), highlighting the importance of uORFs in plant physiology. In some uORF-containing transcripts, such as those for the transcription factor genes *ARABIDOPSIS HOMEOBOX 1* (*AtHB1*) and *BASIC LEUCINE-ZIPPER 11* (*bZIP11*), overproduction of their mORFs leads to deleterious growth phenotypes, suggesting that uORF-mediated suppression plays key roles in regulating plant growth (Ribone et al. 2017). Recently, several studies have manipulated uORFs of genes of interest to improve specific traits. For example, manipulating the uORF in the *bZIP11* transcript increased sugar content in tomato and strawberry (*Fragaria* × *ananassa*; Sagor et al. 2016; Xing et al. 2020), and manipulating the uORF in the *TL1-BINDING FACTOR* (*TBF1*) transcript enhanced pathogen response in Arabidopsis and rice (Xu et al. 2017b).

Using Ribo-seq with improved coverage, 2,093 actively translated uORFs were identified in Arabidopsis seedlings based on statistically significant 3-nt periodicity (Wu and Hsu 2022). However, this approach may not efficiently identify short uORFs or uORFs that partially overlap with their associated mORFs or with other uORFs. Thus, more uORFs likely remain to be identified. Conversely, the number of potential uORFs may be overestimated due to annotation errors. It has been reported that the current Arabidopsis annotation (Araport11) tends to overestimate the length of 5′ UTRs (Parker et al. 2020). If the 5′ UTR length of a transcript is overestimated, a uORF may be predicted from the extended 5′ UTR sequence, which does not exist in nature. A common experiment to validate uORF functions is to compare the 5′ UTRs possessing a wild-type uORF or a mutated uORF (such as by mutating its start codon) in a reporter assay. However, researchers should be cautious, as those false-positive uORFs may still affect translation when artificially expressing non-existing 5′ UTRs. To avoid this issue, researchers can implement 5′ rapid amplification of cDNA ends experiment or exploit available Cap Analysis of Gene Expression-seq data (Takahashi et al. 2012a) to confirm transcription start sites, thereby increasing the confidence in identifying functional uORFs.

The large number of detected uORFs exhibit diverse properties, such as variation in their length and extent of sequence conservation. The smallest possible uORFs are minimum uORFs (AUG-stop; a start codon immediately followed by a stop codon). As translation initiation and termination are the major rate-limiting steps in translation (Shah et al. 2013), translation of minimum uORFs is likely to cause ribosome stalling. The first minimum uORFs reported were identified in Arabidopsis boric acid channel gene *NODULIN26-LIKE INTRINSIC PROTEIN5;1* and in several boron-responsive mRNAs. In response to high boron levels, these minimum uORFs cause strong translational suppression through ribosome stalling and mRNA degradation of these boron transporter and responsive mRNAs, reducing overall boron uptake (Tanaka et al. 2016). Subsequent Ribo-seq experiments have identified the translation of 153 minimum uORFs in Arabidopsis adult leaves, and 119 of these minimum uORFs have increased Ribo-seq levels under high-boron conditions. Interestingly, high boron also leads to global ribosome stalling at the stop codon of mORFs. Therefore, the increase of ribosome footprints at minimum uORFs under high-boron conditions may be partially due to the stalling effects of boron on stop codons (Sotta et al. 2021).

Most identified uORFs lack sequence conservation at the peptide level across plant species, although the presence of uORFs may be conserved between homologous genes. In Arabidopsis, 120 conserved peptide uORFs (CPuORFs) have been identified using bioinformatics approaches (Hayden and Jorgensen 2007; Jorgensen and Dorantes-Acosta 2012; Takahashi et al. 2012b; Vaughn et al. 2012; Van Der Horst et al. 2019; Takahashi et al. 2020). These CPuORFs were further grouped into 81 homology groups based on the peptide sequence similarity of the CPuORFs. This sequence conservation suggests that the CPuORF peptides play important functions. Seven CPuORFs have been reported to interact with specific metabolites (reviewed in Van Der Horst et al. 2020), and 5 CPuORF-encoding peptides have been reported to cause ribosomal arrest, possibly through the interaction between the nascent peptide and the ribosome exit channel (Uchiyama-Kadokura et al. 2014; Hayashi et al. 2017; Yamashita et al. 2017). Interestingly, the peptide sequence of the CPuORF in homology group 30 is similar to CELL DIVISION CYCLE PROTEIN 26 (CDC26) found in fungi and animal species, which is a component of the anaphase promoting complex involved in cell cycle regulation (Takahashi et al. 2012b; Vaughn et al. 2012). A recent study demonstrated that this CPuORF peptide in Arabidopsis indeed acts like CDC26 as a cell cycle regulator, controlling cell division and embryo development (Lorenzo-Orts et al. 2019).

#### Regulation of uORF presence by alternative transcription start sites or alternative splicing

The presence or absence of uORFs in mRNA sequences may be regulated at transcriptional and posttranscriptional levels by altering the length and sequence of 5′ UTRs (Fig. 4B) (Von Arnim et al. 2014). At the transcriptional level, different transcription start sites (TSSs) can influence whether uORFs are included in the mRNA sequences. Some genes were shown to use different TSSs in response to various light conditions, leading to the gain or loss of uORFs in the mRNA sequences (Kurihara et al. 2018). At the posttranscriptional level, an alternative splicing event at the 5′ UTR can determine whether a uORF is present in *HOLOCARBOXYLASE SYNTHASE 1* mRNA, which encodes a protein-biotin ligase that catalyzes the biotinylation of several key metabolic enzymes in Arabidopsis (Puyaubert et al. 2008). Similarly, an intron retention event in the 5′ UTR of *PHYTOCHROME-INTERACTING FACTOR3* (*PIF3*) in response to phytochrome B signaling results in the inclusion of a uORF that represses translation of the *PIF3* mRNA (Dong et al. 2020). It remains an open question whether uORF presence and regulation are widely controlled by alternative TSSs and/or alternative splicing.

#### Regulation of uORF repressiveness

The repressiveness of uORFs can be modulated by stress, phytohormones, nutrients, or metabolites (Fig. 4C).

##### uORF regulation by stress

The translational control of uORFs was first studied in yeast (*Saccharomyces cerevisiae*) and animals, where the translational repression mediated by certain uORFs is alleviated (i.e. derepression), under stress conditions through 2 mechanisms. First, the stringency for uORF initiation can be increased to reduce uORF translation. Second, after the first uORF is translated, a phenomenon called “delayed reinitiation” allows the 40S ribosome to bypass multiple repressive uORFs and reinitiate translation at the mORF (Palam et al. 2011; Young and Wek 2016). Under stress, global translation is typically suppressed to save energy, which is accompanied by selective translation of mRNAs critical for survival (reviewed in Liu and Qian 2014). Therefore, derepression of uORF-containing transcripts provides a way for a rapid and specific increase of certain proteins from the translation of downstream mORFs.

As sessile organisms, fast responses through derepression of uORF-containing genes are likely critical for plants. Two examples of this phenomenon in stress response have been reported. The first example is involved in hypoxia stress (Juntawong et al. 2014). Using Ribo-seq, it was shown that a high fraction of genes with increased translation efficiency under hypoxia contain long uORFs. Additionally, the number of Ribo-seq reads associated with these uORFs decreases under hypoxia, whereas those reads associated with their mORFs increase (Fig. 4C). These results suggest that translation of these long uORFs is reduced, implying that the uORFs are frequently skipped during translation under hypoxia. Alternatively, the reduced translation of uORFs may be due to a global reduction of translation, whereas mORF translation is increased due to enhanced re-initiation.

The second example of derepression involves *TBF1*, which encodes a heat-shock factor-like transcription factor required for immune response. The *TBF1* transcript contains 2 uORFs (Fig. 4C), and the translation of the *TBF1* mORF is de-repressed after pathogen challenge (Pajerowska-Mukhtar et al. 2012). Interestingly, the *TBF1* transcript is decapped during the immune response, and 2 R-motifs (guanine/adenine-rich motifs) in the 5′ UTR of *TBF1* can serve as Internal Ribosome Entry Sites (IRESs) (see section on ‘IRES’ below) to facilitate the translation of *TBF1* mORF. Notably, the 2 R motifs in the *TBF1* mRNA overlap with or are downstream of the uORFs. Therefore, the IRES sequences can allow ribosomes to avoid the uORFs and directly proceed to the *TBF1* mORF (Wang et al. 2022). Whether this IRES-mediated uORF skipping mechanism allows the derepression of other uORF-containing transcripts in different stress conditions in plants is an exciting direction for future research.

##### uORF regulation by phytohormone(s)

The phytohormone auxin can also derepress uORF-containing transcripts by activating the kinase TARGET OF RAPAMYCIN (TOR) (see section ‘Translational regulation through modulation of translation factors and master translational regulators – TOR’ for details). In Arabidopsis, TOR promotes the translational re-initiation of uORF-containing mRNAs by directly phosphorylating eIF3h, a factor known to promote re-initiation. Activated TOR and eIF3h facilitate the polysome loading of several uORF-containing mRNAs, including those of *AUXIN RESPONSE FACTOR*s (*ARF*s) and *bZIP11* (Fig. 4C) (Schepetilnikov et al. 2013). Notably, TOR derepresses uORF-containing transcripts to facilitate growth, while other examples of derepression respond to stress conditions. Because TOR is activated or repressed by many environmental cues or phytohormones, such as light, glucose, nutrients, auxin, and ABA (reviewed in Schepetilnikov and Ryabova 2018; McCready et al. 2020), it will be interesting to learn which genes with uORF-containing transcripts are controlled by TOR under different growth conditions.

##### uORF regulation by nutrients and metabolites

In addition to boron control of minimum uORFs mentioned above (Tanaka et al. 2016), phosphate deficiency facilitates the derepression of several uORF-containing transcripts, including those in *ARF4* and *CYTOKININ RESPONSE FACTOR 10*, which might promote root growth for phosphate uptake (Bazin et al. 2017).

It is well documented that the repressiveness of several CPuORFs is modulated by metabolites. For example, sucrose, thermospermine, and galactinol regulate the activities of the CPuORFs on the mRNAs of the transcription factor genes *bZIP11*, *SUPPRESSOR OF ACAULIS 51*, and *TBF1*, respectively (Rook et al. 1998; Wiese et al. 2004; Imai et al. 2006; Rahmani et al. 2009; Cai et al. 2016; Yamashita et al. 2017; Zhu et al. 2018). Both choline and phosphocholine activate the CPuORF of the *XIPOTL1* transcript, encoding a phosphoethanolamine N-methyltransferase, to repress phosphatidylcholine biosynthesis (Alatorre-Cobos et al. 2012). Interestingly, the biosynthesis and degradation of polyamine are modulated by 2 homology groups of CPuORFs in the transcripts of *S-ADENOSYLMETHIONINE DECARBOXYLASE* (*SAMDC*) and *POLYAMINE OXIDASE*s (*PAO*s), respectively (Hanfrey et al. 2005; Guerrero-González et al. 2014). Finally, under high-ascorbate conditions, a conserved uORF with a noncanonical start site on *GDP-L-galactose phosphorylase* (*GGP*) mRNA suppresses downstream mORF translation (Laing et al. 2015; Hsu et al. 2016). *GGP* encodes a key enzyme for ascorbate biosynthesis. Thus, the suppression of GGP translation by the conserved uORF reduces ascorbate biosynthesis (Laing et al. 2015). The mechanism for uORF-dependent metabolite sensing remains unclear but is possibly due to interactions between the ribosome exit channel, the uORF peptide, and the metabolites (Van Der Horst et al. 2020).

Most of the CPuORFs mentioned above respond to high metabolite levels to repress the translation of their mORFs and thus negatively regulate the biosynthesis of the metabolites, except the CPuORF of *PAO*s. In the presence of high polyamine levels, the *PAO* CPuORF is suppressed, promoting mORF translation for production of PAO for polyamine degradation, whereas the *SAMDC* CPuORF suppresses *SAMDC* mORF translation, preventing the biosynthesis of polyamine. Thus, the 2 groups of CPuORFs act together to balance cellular polyamine levels (Hanfrey et al. 2005; Guerrero-González et al. 2014).

Intriguingly, in contrast to derepression where uORFs are repressive by default, CPuORF33 in the *AtHB1* mRNA is not repressive until activated. *AtHB1* encodes a transcription factor that regulates hypocotyl and leaf development. Several observations suggest that the repressiveness of CPuORF33 requires photosynthetic activity: (1) the uORF is not repressive in dark-grown seedlings; (2) the uORF is repressive only in the aerial tissues of plants grown under light-dark cycles; and (3) chemicals inhibiting chloroplast function abolish CPuORF33 repressiveness, as shown by a reporter assay (Ribone et al. 2017). Together, this finding provides an interesting example of uORF regulation linking the environment and plant development.

#### Why uORF regulation?

Regulating gene expression by uORFs is energy inefficient because these mRNAs are not translated to their full potential. An alternative to producing the same amount of proteins is to generate fewer mRNAs but without uORF repression. Is there an advantage to having uORFs repress translation? Advances on this topic suggest that uORF-mediated regulation offers at least 3 benefits: First, uORFs may provide feedback regulation for maintaining homeostasis of certain nutrients or metabolites (reviewed by Van Der Horst et al. 2020). Second, regulating gene expression at the translational level allows for quick responses to changing environments (Juntawong et al. 2014; Xu et al. 2017b). Finally, translational suppression could be a general mechanism to reduce gene expression noise (see below).

Gene expression is inherently stochastic. Each step of the central dogma, including the synthesis and decay of mRNAs and proteins, fluctuates. This stochasticity results in variance in protein numbers among genetically identical cells, known as gene expression noise (Raj and van Oudenaarden 2008). Recently, using both an experimental approach and mathematical modeling, uORFs of a circadian regulator, *TIMING OF CAB2 EXPRESSION 1* (*TOC1*), were shown to be critical to suppress the gene expression noise of TOC1 and facilitate the homogeneity of the circadian clock in Arabidopsis (Wu et al. 2022). This finding is consistent with prior studies in prokaryotes and other eukaryotes that lower translation can counteract the fluctuation of transcription levels and reduces the variance of protein abundance. Because protein kinases and transcription factors are enriched among uORF-containing genes in plants (Kim et al. 2007; Wu and Hsu 2022), noise reduction for these critical regulators is likely important for stabilizing their regulatory networks despite the associated energy cost.

### Internal ribosome entry site (IRES)

An IRES allows the 43S PIC to bind to the internal region of the mRNA instead of binding to the 5′ cap first (Fig. 3). Besides recent examples in defense mRNAs (Wang et al. 2022; see section on ‘uORFs’ above), another plant gene reported to possess an IRES is *WUSCHEL* (*WUS*; Cui et al. 2015). *WUS* encodes a transcription factor that maintains the stem cell pool in the shoot apical meristem. The IRES-dependent translation of *WUS* transcripts is controlled by La1 RNA-binding protein. La1 is mainly located in the nucleus, but it relocates to the cytoplasm in response to environmental stress, such as UV and the radiomimetic drug zeocin. In the cytoplasm, La1 enhances the translation of *WUS* transcripts, which correlates with the proliferation of stem cells. Plants overexpressing *La1* continue developing true leaves in the presence of zeocin, while wild-type plants cannot. Thus, the regulation of *WUS* by La1 through IRES-mediated translation can control stem cell proliferation in response to environmental stress.

### RNA secondary structure

One recent example of translational control through RNA secondary structures is related to development (Cho et al. 2018). Based on the hypothesis that phloem development is regulated by sucrose, a comparative transcriptome analysis was conducted on poplar (*Populus tremula*) phloem/cambium-specific genes, Arabidopsis herbaceous phloem/cambium-specific genes, and Arabidopsis sucrose-upregulated genes. One gene that met all 3 criteria encodes an RNA-binding protein named JULGI (“stream” or “plant shoot” in Korean; JUL). A screen using a random single-stranded RNA library revealed that JUL preferentially binds to G-rich sequences and G-quadruplex. A search for phloem-specific mRNAs with potential G-quadruplex sequences in their 5′ UTRs identified *SUPPRESSOR OF MAX2 1-LIKE4* (*SMXL4*) and *SMXL5* as potential targets of JUL. SMXL4 and SMXL5 are key regulators of phloem differentiation. Follow-up experiments demonstrated that JUL binds to the G-quadruplex region in *SMXL4* and *SMXL5* mRNAs and represses their translation and thus negatively regulates phloem differentiation.

Another example of translational control mediated by RNA secondary structures is related to temperature sensing (Chung et al. 2020). A Ribo-seq experiment comparing Arabidopsis seedlings kept at 17 °C or transferred to 27 °C for 15 minutes identified hundreds of transcripts with enhanced or reduced translation. A subset of transcripts that were translationally upregulated contained a predicted hairpin structure located approximately 30 nt upstream of the start codon. Among these, *PIF7* was a promising candidate, as it is homologous to *PIF4*, a master regulator of the thermosensing pathway. Further experiments revealed that the translation of *PIF7* mRNA is mediated by the hairpin structure responsive to temperature changes in the *PIF7* mRNA and that PIF7 protein levels are positively correlated with hypocotyl growth in response to a warmer temperature, likely through upregulation of auxin biosynthesis genes by PIF7.

### Sequences recognized by RNA-binding proteins

Two independent studies have described an elegant example of translational regulation acting on the 3′ UTR of *EIN3-BINDING F-BOX 1* (*EBF1*) and *EBF2* mRNAs in the ethylene signaling pathway (Li et al. 2015; Merchante et al. 2015). EBF1 and EBF2 are key repressors of the ethylene signaling pathway by targeting the master transcription factors of ethylene-regulated genes for proteasome-mediated degradation. It was a mystery that *EBF1* and *EBF2* mRNAs were upregulated in response to ethylene, but no increased protein levels were detected. In 1 of the 2 studies, Ribo-seq revealed that the translation of *EBF1* and *EBF2* mRNAs is downregulated by ethylene (Merchante et al. 2015). In the second study, it was observed that small RNAs recognizing the *EBF1* and *EBF2* 3′ UTRs overly accumulate in a mutant defective in the 5′-to-3′ decay enzyme *EXORIBONUCLEASE4* (*XRN4*) gene (also named *ETHYLENE INSENSITIVE 5*). Additionally, overexpression of the *EBF1* 3′ UTR fragment results in ethylene insensitivity (Li et al. 2015). Both studies revealed that the translational control of *EBF1* and *EBF2* by ethylene depends on the 3′ UTR of their mRNAs, particularly poly-U elements. Furthermore, EIN2, another signaling protein in the ethylene pathway, binds to the 3′ UTR of *EBF1* and *EBF2* mRNAs and brings them to the processing bodies (P-bodies, see section ‘Stability and spatial dynamic of mRNAs – P-bodies and stress granules’) with the help of NMD factors. Thus, the localization to the P-bodies prevents *EBF1* and *EBF2* mRNAs from being translated (Li et al. 2015; Merchante et al. 2015).

Another example of translational control through 3′ UTR has been discovered in rice panicle development. *FRIZZY PANICLE* (*FZP*) was identified as a quantitative trait locus for grain number per panicle, and it was shown that an increase in *FZP* 3′ UTR length was associated with increased grain number, but the underlying mechanism was unknown (Bai et al. 2017b; Fujishiro et al. 2018). Recently, the presence of a conserved CU-rich element (CURE) was identified in the *FZP* 3′ UTR. The deletion of the CURE in the *FZP* 3′ UTR resulted in an increase in FZP translation levels, without affecting *FZP* mRNA levels. Similar results were observed by overexpressing the *FZP* 3′ UTR fragment, which presumably competes with the endogenous *FZP* 3′ UTR. Furthermore, the CUREs were shown to be bound by POLYPYRIMIDINE TRACT-BINDING PROTEIN 1 (PTB1) and PTB2. Manipulating *PTB1* or *PTB2* expression altered FZP translation and grain number (Chen et al. 2022).

RNA-binding proteins may also act on elements in the 5′ UTR for translational control. LA AND RELATED PROTEINs (LARPs) are conserved RNA-binding proteins in eukaryotes. Plants possess LARP1 and LARP6 families, each encoded by 3 genes (A to C). *LARP6C* is specifically expressed in pollen. Recently, using RNA-immunoprecipitation sequencing followed by motif analysis, LARP6C was discovered to binds to a B-box motif (either U-rich or UC/UCC-rich) within the 5′ UTRs of its target mRNAs. Interestingly, analysis of a reporter construct for its target revealed that LARP6C can switch from a translational activator to a translation repressor when pollen grains transition from dormancy to germination (Billey et al. 2021).

Another example of RNA-binding protein acting on a 5′ UTR to regulate translation is related to phytochrome signaling. Phytochromes are photoreceptors that sense red and far-red light and switch between Pr and Pfr forms. When exposed to red light, the Pr form switches to the Pfr form, which relocates to the nucleus, promoting gene expression for photomorphogenesis (reviewed in Cheng et al. 2021). In the cytosol, the Pfr form also interacts with the RNA-binding protein PENTA1 (PNT1). PNT1 binds to the 5′ UTR of *PROTOCHLOROPHYLLIDE REDUCTASE* (*PORA*) involved in chlorophyll biosynthesis and represses its translation in the presence of Pfr. The inhibition of *PORA* translation is critical for adjusting chlorophyll contents when seedlings transition from far-red light to white light conditions (Paik et al. 2012). The specific mRNA features on *PORA* mRNA that contribute to the translational control remain to be identified.

### Readthrough

A special mechanism of translational control is readthrough at the stop codon. It occurs when a near-cognate tRNA, instead of release factors, binds to the stop codon at the A-site of the ribosome and continues translation. The resulting C-terminal extensions of the proteins may alter the protein subcellular localization, function, or stability.

Interestingly, the first known readthrough example in Arabidopsis is *eukaryotic RELEASE FACTOR 1* (*eRF1*). The *eRF1* 3′ UTR possesses sequence features predicted to be targeted by NMD, including a long 3′ UTR and an intron distantly downstream of the stop codon. It was shown that when eRF1 levels are high, readthrough is unfavored, promoting NMD and reducing *eRF1* mRNA levels. By contrast, when eRF1 levels are low, readthrough is favored, preventing the *eRF1* 3′ UTR from being targeted by NMD. Thus, the *eRF1* expression forms an autoregulatory circuit (Nyikó et al. 2017).

More recently, mining available Arabidopsis Ribo-seq data revealed that 144 transcripts exhibit stop codon readthrough (Sahoo et al. 2022). Among the 3 possible stop codons, UGA is the preferred stop codon for translational readthrough. In contrast to the consensus readthrough context (stop-CARYYA) defined by a previous mutational analysis in *Nicotiana benthamiana* (Skuzeski et al. 1991), the nt immediately upstream and downstream of the stop codons was found to be enriched for A or U in the readthrough cases (Sahoo et al. 2022). Remarkably, among the 144 transcripts with readthrough, 34 encode RNA-binding proteins. Although the biological significance of these readthrough events remains to be determined, it was demonstrated that the readthrough of *CURVATURE THYLAKOID 1B* results in the inclusion of a nuclear localization signal peptide in the encoded protein, causing the new protein to localize to the nucleus, in contrast to the original isoform that localizes in the cytoplasm (Sahoo et al. 2022).

### G-content in the poly(A) tail

Contrary to the common belief that poly(A) tails are composed exclusively of adenosines, work in human cell lines has revealed the existence of non-A residues in poly(A) tails (Chang et al. 2014). Similarly, ∼16% of Arabidopsis poly(A) tails contain at least 1 non-A residue, and the majority of these are guanines. These G residues break the poly(A) tails into multiple A tracts. Genome-wide analysis showed that the G content in poly(A) tails is negatively correlated with the efficiency of PABP binding and translation, without affecting mRNA stability. Consistent with these observations, transcripts with high PABP binding display a significant reduction in translation efficiency in *pabp* mutants (Zhao et al. 2019b).

### Small interfering RNAs (miRNAs and siRNAs)

In addition to mRNA cleavage, small interfering RNAs (miRNAs and siRNAs) can target specific mRNA sequences for translational repression (Brodersen et al. 2008). Consistent with the expected roles of miRNAs in translational repression, Ribo-seq analysis showed that predicted miRNA targets are associated with lower translation efficiency in Arabidopsis and tomato (Liu et al. 2013; Wu et al. 2019).

Once miRNAs are produced, they bind to AGO and form the RNA-induced silencing complex (RISC), which can trigger mRNA cleavage or inhibit translation. miRNA-mediated translational inhibition was shown to occur at the ER through interaction between AGO1 and an integral ER membrane protein, ALTERED MERISTEM PROGRAM1 (AMP1; Li et al. 2013).

More recently, HYPONASTIC LEAVES1 (HYL1), a core component of miRNA processing machinery, was shown to also function as a miRNA-mediated translational inhibitor (Yang et al. 2021). HYL1 is a double-stranded RNA (dsRNA)-binding protein that is dually localized to the nucleus and cytoplasm, but the function of cytosolic HYL1 was unknown. Interestingly, examining the role of cytosolic HYL1 by introducing a construct encoding a nucleus-localized form of HYL1 in a null *hyl1* mutant background revealed that cytosolic HYL1 does not regulate miRNA biogenesis or the cleavage of the target mRNAs. Instead, the target mRNAs accumulate more proteins in the absence of cytosolic HYL1. Further experiments showed that cytosolic HYL1 localized on the ER and associated with AGO1, AMP1, and miRNA-targeted mRNAs. Importantly, HYL1 facilitates AGO1 loading onto polysomes without affecting the distribution of the target mRNAs in the polysome profile. This result suggests that miRNA-mediated translational inhibition occurs after translation initiation.

Recent work showed that siRNAs, specifically 22-nt siRNAs, play a role in translational regulation (Wu et al. 2020). Plants produce 21-, 22-, and 24-nt siRNAs. Although the functions of the 21- and 24-nt siRNAs are well studied, the function of 22-nt siRNAs was largely unknown due to their low abundance. In double mutants lacking either the 5′-to-3′ decay enzyme XRN4, or the 3′-to-5′ mRNA decay enzyme SUPER KILLER2 (SKI2), along with DICER-LIKE 4 (DCL4), i.e. *xrn4 dcl4* and *ski2 dcl4*, high levels of 22-nt siRNAs are produced, particularly from 2 genes encoding nitrate reductases NIA1 and NIA2. Surprisingly, overaccumulation of 22-nt siRNAs does not lead to a decrease in *NIA1* and *NIA2* mRNA levels but instead causes a dramatic reduction in their protein levels. Polysome profile revealed global translational repression as well as specific translational repression of *NIA1* and *NIA2* mRNAs. Consistent with a role for 22-nt siRNAs in translational repression, a mutant defective in siRNA loading to RISC (*ago1-27*) rescues the overaccumulation of 22-nt siRNAs and restored NIA1 and NIA2 protein levels.

### Cis-natural antisense RNAs

Cis-natural antisense transcripts (Cis-NATs) are noncoding RNAs that overlap with protein-coding genes and are transcribed from the opposite DNA strand. Cis-NATs and their mRNA partners are expected to form dsRNA and lead to RNA degradation; however, some cis-NATs were found to increase the translation of their cognate mRNAs (Deforges et al. 2019; Reis et al. 2021). In the case of rice *PHOSPHATE1.2* (*PHO1.2*), instead of long-range base-pairing between the sense-antisense transcripts, both sense (*PHO1.2*) and antisense transcript (*cis-NAT_PHO1.2_*) alone have extensive intramolecular structures. Upon interaction between the sense-antisense transcripts, local structural changes occur and reduce inhibition of a high GC region located ∼350 nt downstream of the start codon, which is associated with translational repression. It was suggested that the interaction between the sense-antisense transcripts leads to increased 80S formation and possibly enhanced translation elongation, resulting in increased *PHO1.2* translation (Reis et al. 2021).

## Translational regulation through modulation of translation factors and master translational regulators

**Figure 5.**
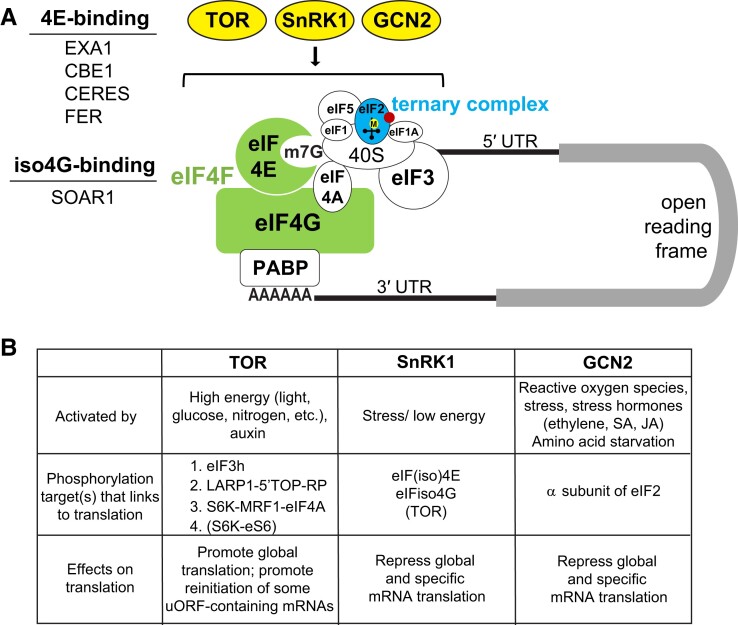
Overview of translational regulation through modulation of translation factors and signaling through conserved master translational regulators. **A)** Two key points of translation initiation are under intensive study: assembly of the eIF4F complex (highlighted in green) and formation of the ternary complex (highlighted in blue). Proteins interacting with and/or phosphorylating the eIF4F complex are listed. The conserved master translational regulators, including TOR, SnRK1 and GCN2, are highlighted in yellow. **B)** Summary of activation, target proteins, and effects on translation of the 3 master translational regulators, TOR, SnRK1, and GCN2. Note that TOR–S6K–eS6 signaling may not have observable effects on translation as new evidence suggests (Dasgupta et al. 2023); SnRK1 also connects to translation through TOR, but currently no evidence supports that TOR is directly phosphorylated by SnRK1.

Translation initiation is the rate-limiting step of translation and is critical for controlling the overall translation rates (Shah et al. 2013). Two points of regulation for translation initiation have been the research focus: (1) recognition of the 5′ cap, which involves the eIF4F complexes; and (2) formation of the ternary complex, which is a component of the PIC (Fig. 5). To date, studies have revealed that both points of regulation are controlled through the phosphorylation of the relevant translation factors. Mass spectrometry and phosphoproteomics have enabled exciting breakthroughs in identifying new factors and regulatory mechanisms involved in translation initiation. Below, we discuss the approaches and advancements in this area. Several newly discovered players directly phosphorylate or interact with core translation initiation factors, such as eIF(iso)4Es and eIF(iso)4Gs. In addition to defining the interactors, recent studies have also extended the upstream or downstream signaling components of 3 conserved master translational regulators, namely TOR, SNF1 RELATED PROTEIN KINASE 1 (SnRK1), and GENERAL CONTROL NONDEREPRESSIBLE 2 (GCN2; Fig. 5).

### Regulation of eIF4F complex assembly through 4E-binding proteins

As eIF4E directly binds to the 5′ cap and recruits eIF4G, which in turn recruits other translation factors, the regulation of eIF4E plays a critical role in translational control, and identifying eIF4E-binding proteins has been of great interest. Most eIF4E-binding proteins in other eukaryotes are translational repressors that compete with eIF4G for binding to eIF4E (Grüner et al. 2018). However, no obvious homologs to yeast or mammalian 4E-binding proteins (4E-BPs) have been identified in plants. Although Arabidopsis LIPOXYGENASE 2 and BASIC TRANSCRIPTION FACTOR 3 have been shown to bind to eIFiso4E in vitro and by yeast 2-hybrid, respectively (Freire et al. 2000; Freire 2005), they do not possess the evolutionarily conserved 4E-binding site (4E-BS: YXXXXLΦ; X being any amino acid and Φ being a hydrophobic amino acid), and their roles in translational control remain unclear.

One eIF4E-binding protein, ESSENTIAL FOR POTEX-VIRUS ACCUMULATION (EXA1), was identified from a mutant screen that showed enhanced autoimmunity (Wu et al. 2017). EXA1 binds to eIF4E as shown by split-luciferase complementation and immunoprecipitation in *N. benthamiana*. EXA1 contains an eIF4E-binding motif as well as a conserved glycine-tyrosine-phenylalanine domain that binds to proline-rich sequences. Consistent with its potential role as a translational repressor, the *exa1* mutant has increased protein levels for several defense-related nucleotide-binding leucine-rich receptors. However, *exa1* does not affect general protein synthesis. How EXA1 specifically regulates a particular set of mRNAs remains to be determined.

A plant-specific eIF4E-binding protein, known as CONSERVED BINDING OF eIF4E1 (CBE1), was identified by searching for the canonical 4E-BS motif among potential cap-binding proteins (Patrick et al. 2018). CBE1 is encoded by a single-copy gene in Arabidopsis and is highly conserved across land plants. CBE1 can form cap-binding complexes in vitro and in vivo. *cbe1* mutant plants are viable but exhibit delayed flowering and upregulated expression of mitosis-related genes. Further investigation is needed to determine the role of CBE1 in translation.

More recently, an eIF4E-binding protein named CERES (named after the Roman goddess of agriculture) was identified through yeast 2-hybrid (Toribio et al. 2019). Like CBE1, CERES is encoded by a single copy gene in Arabidopsis, is specific to plants, and is highly conserved from mosses to angiosperms. CERES interacts with eIF4E via the canonical 4E-binding site, which is also present in eIF4G (Miras et al. 2017). Interestingly, CERES appears to replace the role of eIF4G, forming a translation initiation complex with eIF4E, eIF4A, eIF3, and PABP. Furthermore, in vitro analysis revealed that CERES promotes translation, rather than repressing translation like most 4E-binding proteins in other organisms. CERES also promotes translation of specific mRNAs involved in light response and starch accumulation (Toribio et al. 2019).

In addition to proteins containing the canonical 4E-BS, other proteins may interact with 4E to regulate translation. An interesting regulation of eIF4E controlling local translation is the FERONIA (FER) pathway (Zhu et al. 2020). FER is a receptor-like kinase located at the plasma membrane at the tip of root hair cells. Reanalysis of previous yeast 2-hybrid and immunoprecipitation-mass spectrometry screens revealed that FER interacts with several translation factors involved in initiation and elongation, as well as ribosomal proteins. The interaction with FER sequesters eIF4E to the plasma membrane of root hair tips. Upon binding to its ligand peptide RAPID ALKALINIZATION FACTOR 1 (RALF1), FER undergoes autophosphorylation and triggers eIF4E phosphorylation. The phosphorylated eIF4E moves away from the plasma membrane and binds to the 5′ cap and eIF4G to translate specific mRNAs involved in root hair growth, including those for *ROOT HAIR DEFECTIVE 6-LIKE 4* (*RSL4*). RSL4 is a transcription factor that negatively regulates root hair growth and represses *RALF1* expression. Thus, the RALF1–FER–eIF4E–*RSL4* signaling axis forms a sophisticated negative feedback loop to control local translation and root hair growth. It is worth further investigation to elucidate how the specific translation of *RSL4* mRNA is achieved once eIF4E is released from the plasma membrane. One possibility is that *RSL4* mRNA is also specifically localized at the root hair tip.

Similar to the competition for binding to eIF4E and disrupting eIF4F complex assembly, the RNA-binding protein SUPPRESSOR OF ABAR-OVEREXPRESSOR 1 (SOAR1) interacts with eIFiso4G1 and eIFiso4G2, preventing the assembly of eIFiso4F complexes and repressing translation initiation (Bi et al. 2019). SOAR1 was initially discovered from a suppressor mutant screen in plants overexpressing the putative ABA receptor ABAR (Mei et al. 2014). In addition to eIFiso4G1 and eIFiso4G2, SOAR1 can bind to the mRNA of a key ABA signaling component, *ABA INSENSITIVE5* (*ABI5*). The binding of *ABI5* mRNA releases eIFiso4G1 and eIFiso4G2 from SOAR1 and thus allows the assembly of eIFiso4F complexes and translation initiation (Bi et al. 2019). The mechanisms by which SOAR1 efficiently sequesters high-abundance eIFiso4G1 and eIFiso4G2 and how *ABI5* mRNA binding to SOAR1 releases eIFiso4G1 and eIFiso4G2 remain unclear.

### TOR acts on multiple aspects of translation

TOR is a master translational regulator. TOR belongs to the phosphatidylinositol 3-kinase–related kinase family and is conserved throughout eukaryotes. TOR is regularly reviewed (Schepetilnikov and Ryabova 2018; Shi et al. 2018; Pacheco et al. 2021; Scarpin et al. 2022). Here, we summarize critical knowledge for understanding TOR regulation in translational control and discuss recent advances in the field.

In mammals, TOR regulates at least 3 aspects of translation: (1) translation initiation is regulated by 4E-BPs, which compete with eIF4G for binding to eIF4E. When cells are in a high energy state, 4E-BPs are phosphorylated by TOR, preventing them from binding to eIF4E, which allows the efficient formation of the eIF4F complex and promotes translation initiation. (2) TOR phosphorylates S6 Kinase 1 (S6K1), which in turn phosphorylates the ribosomal protein of small subunit 6, RPS6, or eS6 according to the updated ribosomal protein nomenclature (Scarpin et al. 2023). Note that the significance of eS6 phosphorylation in translation remains controversial (reviewed in Meyuhas 2015). (3) TOR regulates ribosome biogenesis by controlling the translation of ribosomal protein mRNAs through 5′ terminal oligopyrimidine tract (5′ TOP) motifs (reviewed in Burkart and Brandizzi 2021).

In plants, although no sequence homologous 4E-BPs have been identified, the TOR–S6K–eS6 axis is well conserved, and variations of TOR-5′ TOP-ribosomal protein regulation have recently been identified (reviewed in Pacheco et al. 2021; Scarpin et al. 2022). TOR clearly plays an important role in plant translational control, as global polysome levels are reduced in *tor* RNA interference (RNAi) lines or when TOR activity is disturbed (Deprost et al. 2007).

#### Organization of the TOR complex

Most eukaryotes contain 2 TOR complexes (TORC): TORC1 and TORC2. Only TORC1 is conserved throughout eukaryotes (Brunkard 2020). TORC1 is composed of TOR, REGULATORY-ASSOCIATED PROTEIN OF TOR (RAPTOR), and LETHAL WITH SEC13 PROTEIN 8 (LST8; Scarpin et al. 2020). In Arabidopsis, TOR is encoded by a single gene, whereas RAPTOR and LST8 are both encoded by 2 genes (*RAPTOR1* and *RAPTOR2*; *LST8-1* and *LST8-2*; Menand et al. 2002; Mahfouz et al. 2006; Moreau et al. 2012). RAPTOR assists in the recruitment and phosphorylation of TOR substrates, and LST8 binds to the TOR kinase domain, likely regulating TOR activity. Downstream of TOR, Arabidopsis S6K is also encoded by 2 genes (*S6K1* and *S6K2*). Another conserved TOR substrate that regulates translation is TYPE 2A-PHOSPHATASE-ASSOCIATED PROTEIN 46 (TAP46), which is encoded by a single gene (Ahn et al. 2011).

As *TOR* knockout mutants are embryonic lethal, many studies of TOR have used inducible RNAi lines and treatment of TOR inhibitors, such as rapamycin, Torin-1, and the second-generation inhibitor AZD8055 (Deprost et al. 2007; Xiong and Sheen 2012; Xiong et al. 2013). TOR activity is often monitored through the phosphorylation status of S6Ks or eS6.

#### Upstream regulators, interactors, and downstream targets of TOR

TOR senses both intracellular and environmental cues, and its activity is regulated by nutrients (nitrogen, nucleotide, and amino acid availability; Cao et al. 2019; Busche et al. 2021; Liu et al. 2021), energy (glucose, light, and the circadian clock; Xiong et al. 2013; Chen et al. 2018; Li et al. 2019), phytohormones (auxin, brassinosteroids, and ABA; Schepetilnikov et al. 2013; Zhang et al. 2016; Wang et al. 2018; Montes et al. 2022), and stress (Meteignier et al. 2017). Under favorable conditions, TOR promotes translation, growth, and anabolism (Pfeiffer et al. 2016; Li et al. 2017) and suppresses catabolism, such as autophagy (Zhang et al. 2016; Pu et al. 2017).

A phosphoproteomic analysis in response to TOR inhibitors (rapamycin and AZD8055) and a targeted interactome study of TOR complex components (TOR, RAPTOR1, LST8-1) and TOR targets (S6K1, TAP46) revealed an intricate TOR signaling network in plants (Van Leene et al. 2019). In addition to several translation initiation factors, the study determined that CBE1, which interacts with eIF(iso)4E, is regulated and phosphorylated by TOR, suggesting that TOR also mediates the formation of cap-binding complexes in plants (Van Leene et al. 2019). Moreover, parallel profiling of the TOR transcriptome, translatome, proteome, and phosphoproteome placed Arabidopsis LARP1 downstream of TOR in the signaling pathway, which is consistent with other eukaryotes (Scarpin et al. 2020). Together these phosphoproteome studies identified nearly 160 TOR-sensitive phosphoproteins; among them, translation factors and ribosomal proteins were enriched, highlighting the critical role of TOR in translational control (Van Leene et al. 2019; Scarpin et al. 2020, 2022).

Additionally, a suppressor screen performed in the *lst8* mutant background identified YET ANOTHER KINASE 1 (YAK1) as a TOR target. YAK1 interacts with RAPTOR and is directly phosphorylated by TOR (Forzani et al. 2019), which is also supported by phosphoproteomic analysis (Van Leene et al. 2019). More recently, screening mutants with altered hypocotyl elongation in response to TOR inhibition in the dark discovered that EIN2, a key component of ethylene signaling, is also targeted by TOR (Fu et al. 2021). EIN2 can move between the cytoplasm and the nucleus depending on its phosphorylation status, which is controlled by ethylene (Ju et al. 2012; Qiao et al. 2012). Glucose-activated TOR directly phosphorylates EIN2 through sites distinct from those targeted by the canonical ethylene signaling pathway, and the phosphorylated EIN2 cannot enter the nucleus to orchestrate transcription of ethylene-responsive genes (Fu et al. 2021). Because EIN2 can bind to the 3′ UTR of *EBF2* and regulates its translation (Li et al. 2015; Merchante et al. 2015), an interesting question to explore is whether the TOR–EIN2 axis is also involved in translational regulation.

#### Roles of TOR in translational control

##### TOR and eIF3h in uORF-containing mRNAs

As in mammalian systems, eIF3h in Arabidopsis can serve as a scaffold for either TOR or S6K1, depending on TOR activity. When TOR is inactive, S6K1 binds to eIF3h; when TOR is activated by auxin, TOR binds to and phosphorylates eIF3h. Furthermore, the TOR–eIF3h complex is bound to polysomes, which is important for the re-initiation of uORF-containing mRNAs, including that of *ARF*s and *bZIP11*. By contrast, treatment with Torin-1 leads to dephosphorylation of TOR and S6K1, which promotes the formation of the S6K1–eIF3h complex on polysomes. Consistent with this notion, the polysome loading of uORF-containing mRNAs is significantly reduced in *TOR-*RNAi seedlings. These results demonstrate the role of TOR in the translational control of uORF-containing mRNAs (Schepetilnikov et al. 2013).

A small GTPase, RHO OF PLANTS 2 (ROP2), is also involved in auxin–TOR signaling. In response to auxin, ROP2 binds to and activates TOR. Plants with high levels of active ROP2 exhibit high levels of active TOR and S6K1. By contrast, TOR activation by auxin is abolished in *rop2 rop6 ROP4-*RNAi seedlings. ROP2 also determines the localization of TOR in the endosome. It was proposed that the transient binding of ROP2 to TOR in the endosome is associated with TOR activation (Li et al. 2017; Schepetilnikov et al. 2017).

##### TOR and S6K

Several lines of evidence suggest that the TOR–S6K–eS6 pathway is involved in light-enhanced translation: (1) the correlation between eS6 phosphorylation and photosynthesis activity during the light period of the diurnal cycle; and (2) the association of phosphorylated eS6 with polysomes (Turkina et al. 2011; Boex-Fontvieille et al. 2013; Enganti et al. 2018). When comparing Arabidopsis seedlings kept in the dark with those exposed to light, it was shown that light increases the fraction of mRNAs bound to polysomes (Liu et al. 2012). This light-enhanced translation is decreased in the photoreceptor mutant *phyA*, while high translation is maintained in the negative regulator of light-signaling mutant *cop1* in the dark. This finding suggests that light-enhanced translation is under the control of light signaling. In agreement, light-induced eS6 phosphorylation is abolished in the red/far-red and blue light photoreceptor mutants *phyA*, *phyA cry1 cry2*, and eS6 phosphorylation remains high in the light-signaling mutant *cop1* in darkness and in the light. It was further shown that light-induced eS6 phosphorylation is decreased in *TOR-*RNAi seedlings. By contrast, in the *cop1* mutant, TOR signaling remains active in both dark and light conditions and is reduced by a TOR inhibitor or in *TOR-*RNAi seedlings. Together, these data reveal that TOR–S6K1–eS6 is involved in light-enhanced translation, which is critical for photomorphogenesis (Chen et al. 2018).

Note that the biochemical and physiological significance of eS6 phosphorylation remains controversial in yeast and mammals (Meyuhas 2015). New evidence in Arabidopsis shows that an eS6 phospho-deficient variant largely does not affect translation, as shown by polysome profiling (Dasgupta et al. 2023). This negative result is consistent with the findings in yeast using Ribo-seq (Yerlikaya et al. 2016). eS6 phosphorylation may still provide physiological advantages, as plants accumulating the eS6 phospho-deficient isoform showed certain growth defects compared with plants with the eS6 phospho-mimic isoform (Dasgupta et al. 2023).

Additional downstream targets of S6K have been discovered (Lee et al. 2017). Arabidopsis MA3-CONTAINING TRANSLATION REGULATORY FACTORs (MRF1 to MRF4) are homologs of the human tumor suppressor PROGRAMMED CELL DEATH 4, which binds to eIF4A through its MA3 domains, inhibiting eIF4A activity as well as the eIF4A–eIF4G interaction (Loh et al. 2009). Consistent with an expected role in regulating translational initiation, Arabidopsis MRF1 interacts with eIF4A and is associated with ribosomal subunits and monosomes, as revealed by polysome profiling. ^35^S-methionine labeling and polysome profiling analysis showed that *MRF-*RNAi plants exhibit reduced translation under low-energy conditions (dark and starvation) compared with the wild type. Interestingly, *MRF1* expression, MRF1 phosphorylation, and ribosome association are regulated not only by the energy status but also by TOR activity. Furthermore, MRF1 interacts with S6K and is a direct substrate of S6K (Lee et al. 2017).

##### TOR and TAP46

TAP46 is a regulatory subunit of protein phosphatase 2A, which is a downstream target of TOR in yeast and mammals. Arabidopsis TAP46 can also be phosphorylated by TOR. *TAP46-*RNAi plants display similar phenotypes as TOR silencing, including a reduction in global translation, activation of autophagy, and growth arrest. This observation supports TAP46 as a downstream effector of TOR signaling (Ahn et al. 2011). However, how the TOR–TAP46 axis acts on translational regulation remains unclear.

##### TOR–LARP1–5′ TOP

Multi-omics profiling of TOR signaling in Arabidopsis revealed that LARP1 acts downstream of TOR (Scarpin et al. 2020). In mammals, TOR–LARP1 signaling regulates ribosomal protein mRNAs through their 5′ TOP motifs. Analyzing Arabidopsis downstream targets of TOR–LARP1 identified both conserved and plant-specific 5′ TOP mRNAs. Although these 5′ TOP mRNAs do not strictly encode ribosomal proteins, many are related to ribosome biogenesis or translation (Scarpin et al. 2020, 2022).

As near 160 TOR-sensitive phosphoproteins have been recently identified (Van Leene et al. 2019; Scarpin et al. 2020), further characterization of these proteins will facilitate our understanding of the detailed mechanisms of mRNA translational regulation by TOR.

### SnRK1 integrates environmental cues/phytohormones with translational control

SnRK1 is an evolutionarily conserved protein kinase that functions in energy sensing in plants. It is homologous to mammalian AMP-activated protein kinase (AMPK) and yeast sucrose non-fermenting 1 (SNF1). SnRK1, AMPK, and SNF1 are heterotrimeric enzymes that consist of a catalytic subunit (α) with kinase activity and 2 regulatory subunits (β and γ). In Arabidopsis, the α subunit is encoded by 2 genes, *SnRK1*α1 and *SnRK1*α2, which have been the focus of much SnRK1 research (reviewed in Broeckx et al. 2016; Peixoto and Baena-González 2022).

#### SnRK1 interacts with translation factors eIF(iso)4E and eIFiso4G1

Searching for the conserved SnRK1/AMPK/SNF1 binding sites among Arabidopsis translation initiation factors revealed that both eIF4E and eIFiso4E contain 2 SnRK1 consensus binding sites (Bruns et al. 2019). In vitro kinase assays and in vivo interaction studies support the idea that SnRK1α2 directly interacts with and phosphorylates eIF4E and eIFiso4E. Furthermore, SnRK1-mediated phosphorylation of eIF(iso)4E is associated with global translational repression. Consistent with this idea, the reduced polysome formation caused by overexpression of Arabidopsis *SnRK1α2* in yeast could be remedied by a nonphosphorylatable variant of eIF(iso)4E but not by a phosphomimic variant of eIF(iso)4E.

In addition to interacting with eIF4E, a phosphoproteomic analysis comparing wild-type and *snrk1α1* mutant plants under submergence identified eIFiso4G1 as a potential SnRK1 target (Cho et al. 2016). It was further demonstrated that SnRK1 phosphorylates eIFiso4G1 in response to submergence, which, in turn, regulates global translational repression and the specific translation of mRNAs important for hypoxia responses. The *eifiso4g1* knockout mutant and variants lacking phosphorylation are more sensitive to submergence and affect the translation of hypoxia-responsive mRNAs (Cho et al. 2019).

#### SnRK1 integrates ABA signaling and plant growth/stress responses

Recent studies on SnRK1 provide an elegant example connecting the phytohormone ABA, TOR, and plant growth. As the *snrk1α1 snrk1α2* double mutant is not viable, insights into the role of SnRK1α were gained by studying a sesquimutant (*snrk1α1^−/−^ snrk1α2^+/−^*^)^ or inducible knockdown lines (Belda-Palazón et al. 2020; Henninger et al. 2022). Because the sesquimutant shows reduced ABA-mediated growth repression, the role of TOR, a growth-promoting kinase, was examined under ABA treatment. It was shown that SnRK1α, but not SnRK2 (a key player in ABA signaling), directly interacts with TOR; this interaction is enhanced in the presence of ABA. Furthermore, SnRK1α is required for TOR repression, as TOR repression in response to ABA is slower in the *snrk1α* mutant. Under non-ABA treatment conditions, SnRK2 forms a complex with SnRK1 and PROTEIN PHOSPHATASE TYPE 2C (PP2C, a repressor of ABA signaling), promoting growth. By contrast, in the presence of ABA, PP2C binds to ABA and the ABA receptor, thus releasing SnRK1, which represses TOR and reduces growth (Belda-Palazón et al. 2020).

An independent study has shown that, in the presence of ABA, SnRK2 is activated and phosphorylates RAPTOR, which also inactivates TOR, repressing growth. In nonstress conditions, TOR phosphorylates the ABA receptors, repressing the ABA/stress response (Wang et al. 2018). Thus, SnRK1 and SnRK2 coordinately regulate TOR activity, as well as plant growth and stress responses.

More recently, it was shown that SnRK1 changes its subcellular localization from the nucleus to the cytoplasm in response to ABA. Chemicals blocking nuclear export or fusing SnRK1α1 to a nucleus localization signal abolished ABA-dependent TOR repression (Belda-Palazón et al. 2022). SnRK1α is also recruited into stress granules in response to heat stress through interaction with the Tudor staphylococcal nuclease (Gutierrez-Beltran et al. 2021). Thus, the subcellular localization of SnRK1α connects environmental input and downstream signaling responses.

### GCN2 controls ternary complex formation and global translation initiation

Another important and conserved protein kinase involved in translational control is GCN2, which phosphorylates the α subunit of heterotrimeric eIF2. eIF2 plays a unique role in translation initiation as it is part of the ternary complex [eIF2, GTP, Met-tRNA(i)] that carries the initiator tRNA to the 40S ribosome subunit. In yeast and mammals, the formation of the ternary complex requires efficient GDP/GTP exchange on eIF2, with the help of the guanine nucleotide exchange factor eIF2B. Under stress conditions, a highly conserved serine residue in eIF2α is phosphorylated. The phosphorylated eIF2α tightly binds to eIF2B, inhibiting eIF2B-facilitated GDP-GTP exchange. As a result, the available ternary complex for translation is reduced and leads to global translational repression (reviewed in Browning and Bailey-Serres 2015). Unlike the multiple protein kinases regulating eIF2α phosphorylation in other eukaryotes, only 1 protein kinase, GCN2, is known to phosphorylate eIF2α in plants and yeast (Lageix et al. 2008; Zhang et al. 2008).

In plants, eIF2α phosphorylation has been associated with numerous abiotic/biotic stressors and phytohormones, including reactive oxygen species, UV light, cold, salt, wounding, bacterial infection, ABA, ethylene, salicylic acid, and methyl-jasmonate (Lageix et al. 2008; Liu et al. 2019; Lokdarshi et al. 2020b). Consistent with the role of GCN2 activation and eIF2α phosphorylation in translational repression, amino acid or purine starvation, which activates GCN2, represses global translation; this repression is reduced in *gcn2* mutant plants (Lageix et al. 2008; Wang et al. 2017).

However, other evidence has challenged whether eIF2α phosphorylation has a strong inhibitory effect on translation. Plant eIF2 is known to exhibit a weaker affinity for GDP than mammalian eIF2, leading to the suggestion that plant eIF2 GDP/GTP exchange does not necessarily need the help of eIF2B (Browning and Bailey-Serres 2015). Moreover, the conditions that activate eIF2α phosphorylation do not always lead to translational repression or depend on GCN2 (Izquierdo et al. 2018; Lokdarshi et al. 2020a; Zhigailov et al. 2020). For a comprehensive discussion on this topic, see (Lokdarshi and von Arnim 2022). Below, we highlight recent work related to the role of GCN2 in global translational control.

First, transcriptome and polysome profiling analyses were performed in wild-type and *gcn2* plants under a condition that activates GCN2. These analyses uncovered a set of mRNAs that escape translational repression dependent on GCN2. Surprisingly, unlike the findings in yeast and mammals, plant mRNAs differentially regulated by GCN2 are not enriched for specific functions or possess distinct mRNA features, such as uORFs (Lokdarshi et al. 2020a). Further investigation is required to understand how GCN2 specifically regulates the translation of these mRNAs.

A recent study underscores the importance of GCN2 in global translational control in response to submergence (Cho et al. 2022). Upon submergence, ethylene is trapped in the submerged tissues, and it is known that ethylene triggers eIF2α phosphorylation (Lageix et al. 2008), suggesting a role for GCN2/eIF2α phosphorylation in submergence. Under submergence, eIF2α phosphorylation is enhanced and global polysome loading of mRNAs is reduced. To characterize the effect of GCN2/eIF2α phosphorylation and ethylene signaling on ribosome loading of mRNAs, transcriptome and polysome profiling analyses were performed in *gcn2* and *ein2* (defective in ethylene signaling) compared with wild-type plants under submergence. The results revealed that global translational repression is less effective in *gcn2* and *ein2*. Importantly, GCN2 and EIN2 enhance translation of distinct sets of mRNAs under submergence. The data suggest that ethylene activates 2 pathways, 1 dependent on GCN2, and the other 1 dependent on EIN2, to regulate the production of hypoxia-responsive proteins for acclimation to submergence. Furthermore, translation of several hypoxia-responsive mRNAs under submergence is affected in an eIF2α nonphosphorylatable variant in which the conserved serine residue was mutated to alanine, highlighting a role for eIF2α phosphorylation in translational control in response to submergence (Cho et al. 2022).

### Other previously missed types of regulation

#### tRNA-derived fragments

In addition to carrying amino acids to translating ribosomes during translation, tRNAs have other regulatory functions. One interesting function is that tRNA-derived fragments (tRFs) can regulate translation. Most tRFs are derived from mature tRNAs and can be grouped into 2 categories based on their size and where the cleavage occurs within the tRNA: long tRFs ranging 30 to 35 nt are cleaved at the anticodon region, and short tRFs ranging 19 to 25 nt are cleaved at the D or T arms. Analysis of small RNA-seq data has revealed that plants contain a diverse population of tRFs, and their composition fluctuates depending on developmental stages or environmental conditions (Cognat et al. 2017). Testing 10 synthesized tRFs has shown that 2 of them, derived from tRNA^Ala^(AGC) and tRNA^Asn^, strongly repress the translation of a reporter mRNA in vitro. Interestingly, this translational repression does not require sequence complementarity between the tRF and the translated mRNA. Furthermore, it was shown that the tRFs are associated with active polysomes but not efficiently with ribosome subunits. Thus, tRFs are unlikely to interfere with the binding of ribosome subunits to the mRNA (Lalande et al. 2020). The mechanism by which tRFs repress translation remains to be resolved.

#### A tRNA^His^ guanylyltransferase

Another regulation related to tRNA was discovered serendipitously from a genetic screen searching for a thermosensitive mutant in rice, identifying the mutant *adaptation to environmental temperature 1* (*aet1*) (Chen et al. 2019). *AET1* encodes a tRNA^HIS^ guanylyltransferase, which modifies pre-tRNA^HIS^ and is required for tRNA maturation and homeostasis. Interestingly, yeast 2-hybrid screening for AET1-interacting partners revealed that AET1 interacts with the ribosome-associated protein RECEPTOR FOR ACTIVATED C KINASE 1 A and eIF3h. The interaction was confirmed in planta, and it was demonstrated that all 3 proteins localize to the ER. To investigate a potential role for AET1 in mRNA translation, RNA-immunoprecipitation sequencing was performed, and *OsARF* mRNAs were highly enriched. As discussed above, Arabidopsis *ARF* mRNAs harbor uORFs and the re-initiation of their mORFs is regulated by eIF3h (Schepetilnikov et al. 2013). Consistent with the potential role of AET1 modulating translation re-initiation through eIF3h interaction, the fraction of *OsARF19* and *OsARF23* mRNAs in active polysomes is reduced in *aet1*.

#### eEF2

During translation elongation, eukaryotic Elongation Factor 2 (eEF2) regulates the transfer of peptidyl tRNA from the ribosomal P-site to the A-site. A post-translational modification on a histidine residue of eEF2, termed diphthamide (a target of diphtheria toxin in humans), is conserved in Arabidopsis (Zhang et al. 2022). Arabidopsis mutants of Dph1, which catalyzes the first step of diphthamide biosynthesis, lose the modification on the H700 residue and increase −1 frameshift errors, as shown by a reporter assay. The mutant also displays a decrease in TOR activity, cell proliferation, and plant growth and is accompanied by an increased autophagy. Interestingly, in response to copper and cadmium, wild-type plants accumulate the eEF2 form with unmodified H700 residue, suggesting that an alteration of translational fidelity is involved in acclimating to heavy metal stress.

## Stability and spatial dynamic of mRNAs

In the cytosol, mRNAs might be subjected to degradation through various mechanisms or stored in RNA-protein conjugates, including P-bodies and stress granules. Both degradation and storage can prevent mRNAs from being translated.

### Decay of mRNAs

Most mRNA degradation is initiated by removing the 3′ poly-A tail, followed by either 5′-3′ or 3′-5′ decay mechanisms. The 5′-3′ decay is governed by the decapping complex and 5′-3′ exonuclease XRN4, and 3′-5′ decay is governed by SUPPRESSOR OF VARICOSE or the RNA exosomes (reviewed in Brunkard and Baker 2018; Sieburth and Vincent 2018). Eukaryotic cells also possess 3 conserved translation-dependent RNA quality control mechanisms to remove aberrant RNAs: NMD, no-go decay, and non-stop decay. NMD recognizes mRNAs containing a premature stop codon, no-go decay targets mRNAs with stalled ribosomes, and non-stop decay recognizes mRNAs lacking a stop codon (reviewed in Powers et al. 2020). In plants, NMD is the best-characterized mechanism among the 3 (reviewed in Ohtani and Wachter 2019), whereas no-go decay and non-stop decay have just started to be characterized (Szádeczky-Kardoss et al. 2018a, 2018b).

It was long believed that mRNAs were either engaged in translation, or subjected to degradation or storage. However, prevalent co-translational decay was reported in yeast (Hu et al. 2009; Pelechano et al. 2015). Consistent with this idea, heat induces the accumulation of several decapped mRNAs in light polysome fractions in the Arabidopsis *xrn4* mutant, supporting the notion that some mRNAs are subjected to degradation while bound by ribosomes (Merret et al. 2015). Furthermore, genome-wide analysis of RNA degradation intermediates in wild-type Arabidopsis plants detected a 3-nt periodicity within the coding regions of mRNAs, a signature of actively translating ribosomes, indicating that the transcripts are also occupied by translating ribosomes when degradation occurs (Hou et al. 2016; Yu et al. 2016). Comparative analysis of 10 angiosperm species confirmed that co-translational decay is widespread and conserved across diverse plant lineages (Guo et al. 2023a).

### P-bodies and stress granules

P-bodies and stress granules are cytosolic RNA-protein assemblies that play an important role in balancing mRNA translation, storage, and degradation. Unlike P-bodies, which are constitutively present in cells, stress granules are induced by stress conditions. These topics are reviewed regularly, for example, (Chantarachot and Bailey-Serres 2018; Kearly et al. 2022). Here, we focus on their functions in translational control.

P-bodies were thought primarily to be involved in mRNA degradation, as they are associated with decapping enzymes and the 5′-3′ exonuclease XRN4. However, a recent study has highlighted the storage capacity of P-bodies. As mentioned above, light can enhance the translation of many transcripts (Liu et al. 2012; Chen et al. 2018). Monitoring P-body accumulation in seedlings kept in the dark or transferred to light for 4 h revealed that light reduces P-body accumulation. Furthermore, transcriptome and translatome analysis comparing a mutant defective in P-body formation (*dcp5-1*, a weak mutant allele of *DECAPPING 5*) and wild-type seedlings identified over 2,000 mRNAs that remain actively translated in *dcp5-1* in the dark without changes in their mRNA abundance. These results suggest that in the dark, plants repress the translation of specific mRNAs involved in the translation machinery, photosynthesis, and apical hook formation by transiently storing these mRNAs in P-bodies (Jang et al. 2019). Similarly, in response to hypoxia, OLIGOURIDYLATE BINDING PROTEIN 1 (UBP1) can sequester thousands of mRNAs into stress granules, preventing them from being translated. Importantly, once the stress is removed, these mRNAs reassociate with polysomes to resume active translation (Sorenson and Bailey-Serres 2014).

*EBF2* mentioned above is also an elegant example of P-body regulation of mRNA localization. When the 3′ UTR of *EBF2* is bound by EIN2-C (the C-terminal fragment of EIN2, following cleavage by a protease in response to ethylene signaling), the mRNA is targeted to P-bodies with the assistance of NMD factors (Li et al. 2015; Merchante et al. 2015).

A recent exciting study linked P-bodies, NMD, and translational control (Cairo et al. 2022). *THREE-DIVISION MUTANT 1* (*TDM1*) is specifically expressed during the second meiotic division of pollen development and is required for the termination of meiosis. TDM1 was shown to be recruited into P-bodies by interacting with SUPPRESSOR WITH MORPHOGENETIC EFFECTS ON GENITALIA 7 (SMG7), an NMD factor. A suppressor mutant screen in *smg7-6* background and a yeast 2-hybrid screen for TDM1-interacting proteins led to the identification of canonical eIF4G, as well as the plant-specific eIFiso4G1 and eIFiso4G2, as additional factors involved in meiotic exit. Follow-up experiments revealed that TMD1 recruits eIF(iso)4Gs and 4A into P-bodies, suggesting that translation is temporarily repressed at meiotic exit by moving these translation factors into P-bodies. Consistent with this idea, treating *tmd1* mutants with the translation inhibitor cycloheximide rescues the pollen meiotic exit defect. Thus, reduced translation of meiotic mRNAs is proposed to be critical for completing pollen meiosis.

## Conclusions

Technological advancements have offered unparalleled opportunities to discover noncanonical translation events and dissect the detailed mechanisms of translational control in various aspects of plant development and physiology. As our understanding of translational control still lags behind that of transcriptional control, ample exciting opportunities for new discoveries are within reach.

## Dive Curated Terms

The following phenotypic, genotypic, and functional terms are of significance to the work described in this paper:

TAP46 Gramene: AT5G53000
TAP46 Araport: AT5G53000

ROP2 Gramene: AT1G20090
ROP2 Araport: AT1G20090

NIA1 Gramene: AT1G77760
NIA1 Araport: AT1G77760

NIA2 Gramene: AT1G37130
NIA2 Araport: AT1G37130

PTB1 Gramene: AT3G01150
PTB1 Araport: AT3G01150

PTB2 Gramene: AT5G53180
PTB2 Araport: AT5G53180

RPS6 Gramene: AT4G31700
RPS6 Araport: AT4G31700

WUS Gramene: AT2G17950
WUS Araport: AT2G17950

ARF4 Gramene: AT5G60450
ARF4 Araport: AT5G60450

MAX2 Gramene: At2g42620
MAX2 Araport: At2g42620

TAS1c Gramene: AT2G39675
TAS1c Araport: AT2G39675

HYL1 Gramene: AT1G09700
HYL1 Araport: AT1G09700

GCN2 Gramene: At3g59410
GCN2 Araport: At3g59410

EIFISO4G1 Gramene: AT5G57870
EIFISO4G1 Araport: AT5G57870

AMP1 Gramene: AT3G54720
AMP1 Araport: AT3G54720
